# Modelling dominance in a flexible intercross analysis

**DOI:** 10.1186/1471-2156-10-30

**Published:** 2009-06-28

**Authors:** Lars Rönnegård, Francois Besnier, Örjan Carlborg

**Affiliations:** 1 Statistics Unit, Dalarna University, Borlänge, Sweden; 2 Department of Animal Breeding and Genetics, Swedish University of Agricultural Sciences, Uppsala, Sweden

## Abstract

**Background:**

The aim of this paper is to develop a flexible model for analysis of quantitative trait loci (QTL) in outbred line crosses, which includes both additive and dominance effects. Our flexible intercross analysis (FIA) model accounts for QTL that are not fixed within founder lines and is based on the variance component framework. Genome scans with FIA are performed using a score statistic, which does not require variance component estimation.

**Results:**

Simulations of a pedigree with 800 *F*_2 _individuals showed that the power of FIA including both additive and dominance effects was almost 50% for a QTL with equal allele frequencies in both lines with complete dominance and a moderate effect, whereas the power of a traditional regression model was equal to the chosen significance value of 5%. The power of FIA without dominance effects included in the model was close to those obtained for FIA with dominance for all simulated cases except for QTL with overdominant effects. A genome-wide linkage analysis of experimental data from an *F*_2 _intercross between Red Jungle Fowl and White Leghorn was performed with both additive and dominance effects included in FIA. The score values for chicken body weight at 200 days of age were similar to those obtained in FIA analysis without dominance.

**Conclusion:**

We have extended FIA to include QTL dominance effects. The power of FIA was superior, or similar, to standard regression methods for QTL effects with dominance. The difference in power for FIA with or without dominance is expected to be small as long as the QTL effects are not overdominant. We suggest that FIA with only additive effects should be the standard model to be used, especially since it is more computationally efficient.

## Background

Large genetic differences between founder breeds are utilized in experimental crosses of outbred lines, which gives a high power of detecting quantitative trait loci (QTL) even for moderately sized pedigrees. The commonly used regression model to detect QTL assumes a biallelic QTL fixed within each of the two founder lines [1]. Most traits have a substantial within-breed heritability and we may therefore expect that some QTL are not fixed. If the QTL is not fixed within founder lines, the regression model will underestimate the QTL effect and the power to detect the QTL decreases [2]. In an earlier paper [3] we developed a flexible intercross analysis (FIA) to enhance the detection of QTL in experimental crosses of outbred lines. FIA is a variance component based model which is able to detect QTL at different degrees of fixation within founder lines. Genome scans are performed based on a score statistic in FIA, which gives a computationally efficient and statistically powerful method since it does not require estimation of variance components. The model is also flexible because it can be applied on advanced intercross lines with an arbitrary number of generations. We have shown that the power of FIA is similar to Haley-Knott (HK) regression [1] for fixed QTL and FIA is superior to HK-regressions for QTL that are not fixed within founder lines. We also showed that the differences between FIA and HK-regression is larger for pedigrees with small base generations than for pedigrees with large ones. However, the model was developed and tested for additive QTL only.

Other methods have previously been developed to account for within-line QTL variation. Most of these methods do not include dominance effects (e.g. [4]). Two exceptions are Knott et al. [5] and Pérez-Enciso et al. [6]. Knott et al. [5] developed a nested within half-sib family model that does not assume fixation of QTL alleles in the founder lines, and the number of alleles is only constrained by the number of families. This model was further developed by Kim et al. [7] for analysis of *F*_2 _intercrosses and includes both line effects and half-sib family effects. Dominance is estimated in the line effect whereas the family effect is an overall allele substitution effect. This is a model specifically designed for *F*_2 _intercrosses with fixed effects only and the number of estimated parameters increases with the number of half-sib families. Furthermore, the genotypic information of the dams is not included in the model and the sires are assumed to be unrelated. Pérez-Enciso and Varona [2] developed a mixed QTL model that accounts for line differences and within-line variation of QTL effects. In this model, which is similar to the model developed by Wang et al. [4], a fixed line effect is estimated together with a random within-line QTL variance. This model was further extended to include dominance in Pérez-Enciso et al. [6]. A drawback of the model is, however, the difficulty to compare estimates in different genomic locations as the total QTL variance is a combination of fixed and random effects. The method is also slow since it utilizes a derivative-free method to maximize the log-likelihood in each tested chromosome position. There is therefore a need to develop a method which is computationally efficient, includes dominance and can be applied on general pedigrees from line crosses. We may expect major genes to have considerable dominance effects [8] but this does not necessarily imply that the power of a QTL analysis will increase by including dominance effects in the statistical model. In a recent paper by Martinez [9], the power to detect a QTL having a dominance effect using a variance component (VC) model was studied. He found that the gain in power using a model with both additive and dominance effects was not substantial compared to a model with only additive effects as long as the QTL effect was not overdominant. In the simulation study performed by Martinez, non-inbred full-sib families were simulated and all founder QTL allele effects were assumed to be independent. FIA is a variance component based method which models dependencies between founder QTL allele effects. This difference between FIA and the model studied by Martinez [9] implies that Martinez' results cannot be directly applied on FIA.

The aim of this paper is to extend the FIA model to include both additive and dominance effects, where this extended version is computationally efficient and possible to apply on general pedigrees from line crosses. This version of FIA is then used to test the importance of including dominance in terms of power for QTL detection. We compare the power of the model, by means of simulations, with the original version of FIA and HK-regression. The model is also applied on chicken body weight at 200 days of age in an *F*_2_-cross between wild Red Jungle Fowl and domestic Leghorn. The HK-regression model was chosen for comparison in our simulations because the assumptions of the model are simple and also because it is extensively used in QTL analysis (e.g. [1,10,11]).

## Results and discussion

### Simulation results for a QTL with additive and dominance effects

**Figure 1:**
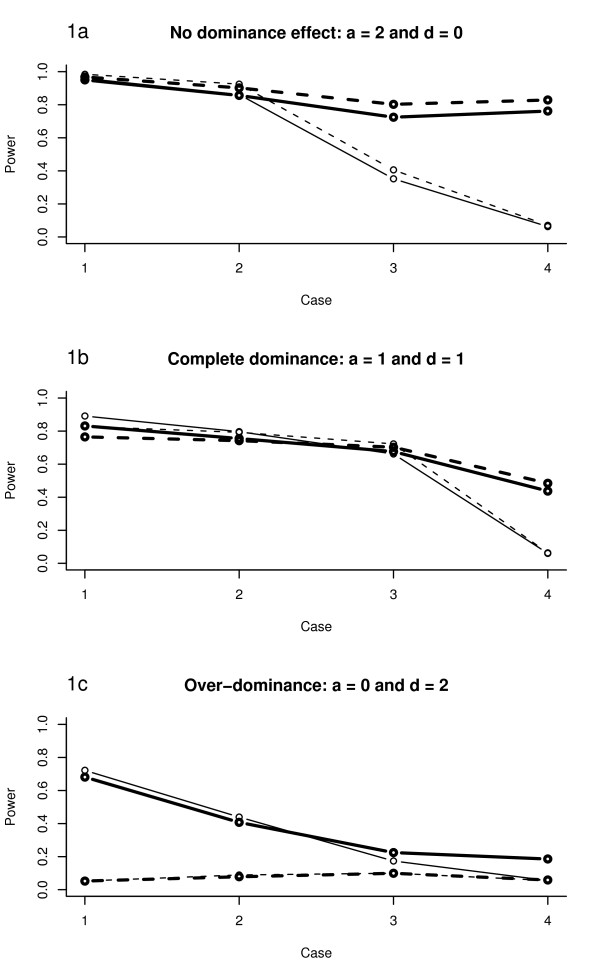
**Power analysis for a simulated QTL**. The power to detect a QTL at a 5% significance level with Haley-Knott (HK) regression and FIA for the four simulated cases presented in Table 1, ranging from total fixation (Case 1) to equal allele frequencies in both lines (Case 4). Thick solid line – FIA with additive and dominance effects; thick dashed line – FIA with additive effects only; thin solid line – HK-regression with additive and dominance effects; thin dashed line – HK-regression with additive effects only. For each case, 6000 replicates were simulated and the pedigree in each replicate had four founders and 800 *F*_2 _individuals. In Figure 1a, an additive QTL effect (*a*) of 2 and a QTL dominance effect (*d*) of 0 was simulated together with a residual variance of 98. In Figure 1b, *a *= 1 and *d *= 1, and in Figure 1c, *a *= 0 and *d *= 2.

The performance of FIA and HK-regression was studied for a simulated QTL with no dominance (Figure 1a), complete dominance (Figure 1b) and overdominance (Figure 1c). Furthermore, four different cases (Table 1) were studied by varying the fixation level within lines for a biallelic QTL. The results show for no simulated dominance that the difference in power between FIA and HK-regression increases when the difference in allele frequency between founder lines increases (Figure 1a). The power of FIA with only additive effects included in the model is higher than FIA with both additive and dominance effects included. These results are very similar to the ones found in Rönnegård et al. [3]. For complete dominance (Figure 1b), we can see that FIA with only additive effects performs just as well as FIA with both additive and dominance effects included. The difference in power between FIA and HK-regression is not as large as in Figure 1a but there is still a large difference when there are equal allele frequencies within founder lines, i.e. for Case 4. For the simulations with extreme overdominance, the power of FIA with only additive effects is approximately 5%, i.e. what we can expect to find by chance alone. FIA with dominance effects included performs better than FIA with only additive effects, and the differences between FIA and HK-regression are small. It should also be noted that our simulations indicate that the difference in power for HK-regression with or without dominance included is also small as long as the QTL effects are not overdominant.

**Table 1:** Simulated levels of fixation for the four simulated scenarios ranging from a fixed QTL (Case 1) to equal frequencies in both founder lines (Case 4)

		Case 1	Case 2	Case 3	Case 4
Line A	Proportion A alleles	1	1	3/4	1/2
Line A	Proportion B alleles	0	0	1/4	1/2
Line B	Proportion A alleles	0	1/6	1/4	1/2
Line B	Proportion B alleles	1	5/6	3/4	1/2

### QTL genome scan for body weight in the Red Jungle Fowl × White Leghorn *F*_2 _Cross

**Figure 2:**
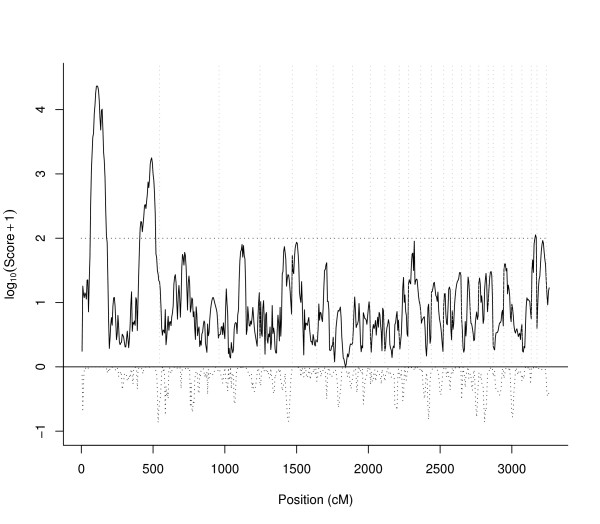
**Genome scan for body weight at 200 days of age**. Genome scan with score values on a *log*_10 _scale. The solid curve above 0 show the score values for FIA including both additive and dominance effects. The dashed curve below 0 show the difference in *log*_10 _score values to those obtained from FIA with only additive effects. The 5% genome-wide significance level is shown as a dashed horizontal line and the borders between chromosomes are given as vertical dashed lines. The score statistic of the FIA model is non-negative since it is defined as a quadratic form.

The chicken genome was scanned for QTL affecting body weight at 200 days of age in an *F*_2 _intercross between Red Jungle Fowl and White Leghorn. As previously [3,11] reported there are two QTL with large effects on chromosome 1. These two QTL give very large score values in our study also (Figure 2) and the peak values are far above the 5% genome-wide significance threshold of 101.2. The significance threshold for the same data without dominance effects included in FIA was 85.6. This increase in threshold value is expected since more parameters are included in FIA with dominance. The changes in score values in the genome scan are relatively small (Figure 2) and there is only one more peak that exceeds the significance level of 101.2. This QTL is located on chromosome 27 (i.e. the third chromosome from the right in Figure 2). There are also several suggestive QTL located on chromosomes: 3, 4, 5, 11 and 28. The only one of these suggestive QTL that showed a substantial change in the score value after including dominance effects in FIA was the QTL on chromosome 4. In conclusion, the change in score values was small for FIA with or without dominance effects and the significance of the QTL were mainly affected by the difference in the genome-wide significance threshold between the two models.

### What do the results tell us about the importance of including dominance effects in FIA?

Our simulations show that the power of FIA including dominance effects is substantially higher for overdominant QTL. For QTL effects that are not overdominant the differences between the two versions of FIA are small. Hence, it is feasible to include dominance in FIA. We expect, however, that major genes having moderate dominance effects will be detected with the simpler additive version of FIA. These results are similar to the ones obtained by Martinez [9] where he showed that the power of VC-based models does not increase substantially by including dominance effects as long as the QTL effects are not overdominant. The difference in power for HK-regression with or without dominance included in the model seem to be small as long as the QTL effects are not overdominant. So the importance of including dominance effects in QTL analysis seems to be a general question and is related to how often we can expect major genes to be overdominant.

Although the differences between HK-regression and FIA decreases for dominant QTL effects we still have not found a case where HK-regression outperforms FIA substantially in terms of QTL detection power. Regression methods are computationally faster than FIA although the latter is based on the score statistic which is easily computed. For the simulated pedigree with 800 *F*_2 _individuals, including dominance in FIA gives a three-fold increase in computational costs (wall clock-time) for the score statistic (eq. 12).

Including dominance also requires that the dominance IBD-matrices have been computed, which may be computationally demanding unless the IBD calculations are based on the gametic IBDs (see eq. 3). The genome scan in FIA is based on a score statistic (eq. 12) and the variance components in FIA do not need to be estimated for each position, but for QTL positions we may wish to estimate the variance components of FIA. There are then two variance components for the additive effects, two for the dominance effects (see eq. 11) and one for the residual variance. Although the VC estimates are of secondary importance in FIA, estimates of the five variance components in eq. (11) are given in the Appendix for each of the four cases in Table 1, for 120 replicates of the simulated 800 *F*_2 _pedigree. Models with several variance components require a robust REML estimation algorithm to ensure convergence. Mishchenko et al. [12] recently developed a robust and efficient REML estimation algorithm for VC models including up to five variance components, which was not applied in our current study but is likely to become useful in the future.

We have previously shown that it is computationally feasible to include epistasis in FIA [3] but so far we have not tested FIA with epistasis on empirical data, and we may expect HK-regression to be a useful method for detection of epistatic QTL effects (e.g. [10]) still for some time in the future. We are convinced that an important research task is to develop a computationally fast and robust version of FIA for detection of epistatic effects.

## Conclusion

We have shown that FIA can be extended to include QTL dominance effects. The power of FIA is superior, or similar, to HK-regression for QTL effects with dominance. The difference in power for FIA with or without dominance is small as long as the QTL effects are not overdominant. Furthermore, we expect that FIA with only additive effects included will be effective also for finding major genes having moderate dominance effects. We therefore suggest that FIA with only additive effects should be the model to use in most situations especially since it is computationally less intensive.

## Methods

In this section we present the traditional single locus VC model that includes dominance effects of the QTL and where all base QTL allele effects are assumed to be uncorrelated [13,14]. Thereafter, we present our FIA model which was previously developed for additive QTL effects [3] and show how dominance can be included.

### Traditional VC model including dominance QTL effects

The VC model including QTL effects with dominance is given by:

(1)

where *y *is the vector of individual phenotypes (length *n*), *b *is a vector of fixed effects and *X *is the corresponding design matrix, *v *is a vector of additive random individual QTL effects (length *n*) in position *τ*, *d *is a vector of random individual QTL effects for dominance (length *n*), and *e *is a vector of residual effects (length *n*). The variance-covariance matrix of *y*, assuming independent allelic effects in the base generation, is (e.g. [15]):

(2)

where Π is the genotype IBD-matrix (size *n *× *n*) calculated in position *τ*,  is the corresponding genotype QTL variance for additive effects, Δ is the dominance IBD-matrix (size *n *× *n*) calculated in position *τ*,  is the QTL variance for dominance effects, *I *is the identity matrix of size *n *× *n*, and  is the residual variance. An element in row *i *and column *j *of Δ can be calculated directly from the gametic IBD-matrix (e.g. [16]) as:

(3)

where the values *g*_*ij*_(*k*, *l*) are the gametic IBDs between individual *i *and *j *for the maternal/paternal alleles *k *and *l*.

### Including dominance in the VC QTL model

Rönnegård and Carlborg [17] described the VC model in eq. 1 in terms of independent base generation effects, where:

(4)

Here v* is a vector of base generation allele effects and d* is a vector of dominance effects for all pairwise base allele combinations. These dominance effects are assumed to be randomly sampled from an infinite population of dominance effects with a variance of . Furthermore the random dominance effects for homozygotes and heterozygotes are assumed to be sampled from the same distribution. The incidence matrices *Z *and *W *relate individuals with their corresponding additive and dominance effects. We thereby have a variance-covariance matrix for the random effects given by:

(5)

Moreover, with this notation we have the relationships (see [17])

(6)

(7)

Hence, for a single QTL model there is no covariance between additive and dominance effects. The estimates of  and  may be strongly correlated, however, since the IBD-values in Π and Δ are correlated [9].

### FIA model with additive effects

FIA extends the traditional VC model to include within-line correlations of the QTL allele effects. The FIA model without dominance effects is given by [3]:

(8)

where the variance-covariance matrix of *y *is:

(9)

Here, Π_*I *_is the genotypic IBD-matrix assuming independent QTL allele effects in the base generation and Π_*J *_is the IBD-matrix that assumes fixation of QTL alleles within founder lines. Hence, the analysis using FIA requires an IBD estimation program that allows for different base generation structures. We used the same IBD-matrix estimation program as in [3], which is based on the deterministic algorithm published by [16].

### FIA model with additive and dominance effects

Dominance is included in FIA by using the same linear model as in (1) but the variance-covariance matrix is not the same as in (2):

(10)

where the variance-covariance matrix of *y *is:

(11)

Here, Δ_*I *_is the dominance IBD-matrix assuming independent QTL allele effects in the base generation and Δ_*J *_is the dominance IBD-matrix that assumes fixation of QTL alleles within founder lines. The above formula for the variance-covariance matrix *V *was derived following the derivation of eq. (4) in Rönnegård et al. [3].

We let the variance components be independent of each other. This assumption gives the variance-covariance matrix of *y *as a linear function of the variance components. This is a simplification since  is the same within-line correlation as  and the variance-covariance matrix of *y *is not strictly a linear function of the variance components.

### Calculating the score for the FIA model

FIA utilizes the score statistic [18-20]

(12)

where *D *is the gradient and *F *is the information matrix calculated under the null hypothesis of no QTL effects, i.e. .

The elements of the gradient *D *of the log-likelihood function *L *are given by [21]:

(13)

where  and . The partial derivatives of *V *are: , and . Furthermore, *P *is the projection matrix given by:

(14)

The elements of the information matrix *F *are given by [21]:

(15)

### Calculation of genome-wide significance thresholds

The significance thresholds for the genome scan were calculated by means of permutation testing (as in [3]). Residuals were calculated from a null model assuming no QTL effect. These residuals were then permuted giving a new vector *ĕ*. Replicates of the phenotypic data were simulated with  where  is the vector of fixed effects estimated from the null model *y *= *Xb *+ *e*. For each replicate, the score statistic was calculated at every tested position (5 cM apart) along the genome using 12. The empirical distribution of the maximum score value from each replicate was used to obtain significance thresholds. 2000 replicates were simulated.

### Simulation setup

In the power analyses, level of fixation within founder lines and degree of dominance were varied to evaluate the differences between FIA and HK-regression. The methods were compared by their power to detect a QTL at a given position at a 5% significance level.

The structure for the base generation was designed to mimic the pedigree of a Red Jungle Fowl – White Leghorn *F*_2 _Cross [11] with one Jungle Fowl male mated to three Leghorn females, and 800 *F*_2 _individuals. Four different cases (Table 1) were studied by varying the fixation level within lines for a biallelic QTL. The QTL was simulated at a position having a fully-informative marker so that the QTL alleles could be traced through the pedigree unambiguously.

The phenotype of an *F*_2 _individual *i *was simulated with *y*_*i *_= *A*_1*i *_+ *A*_2*i *_+ *D*_*i *_+ *e*_*i *_where *A*_1*i *_is the QTL allele effect on the paternally inherited chromosome and *A*_2*i *_is the QTL allele effect on the maternally inherited chromosome, *D*_*i *_is the dominance effect and *e*_*i *_is an iid normally distributed residual effect with a variance equal to 98. A biallelic QTL was simulated where the additive effects for the two alternative alleles were 0 and *a*, and the dominance effects for heterozygotes was *d*. The values of *a *and *d *were varied from 0 to 2.

6000 replicates were calculated for each of the four cases in Table 1 and for varying degrees of dominance.

### Analysis of experimental data: Red Jungle Fowl × White Leghorn *F*_2 _Cross

In a Red Jungle Fowl × White Leghorn F2 cross, we performed a full genome scan using FIA with additive and dominance effects. In this pedigree, one Red Jungle Fowl male was mated to three White Leghorn females producing 756 *F*_2 _offspring with measured genotypes and phenotypes. We used an updated marker map to those reported in [11] including 439 markers (Leif Andersson, personal communication) covering chromosomes 1 to 28. We analyzed body weight at 200 days of age. In our previous study using FIA with only additive effects we found six QTL at a 5% genomwide significance. These QTL were located at: 102 cM on chromosome 1, 488 cM on chromosome 1, 32 cM on chromosome 5, 30 cM on chromosome 6, 21 cM on chromosome 27 and 35 cM on chromosome 28. The data are described in detail in [11].

## Authors' contributions

LR performed most of the analysis and writing. FB calculated the IBD-matrices and added important ideas to the text. ÖC initiated the paper and was responsible for the development of the paper from the initial results to the final version of the manuscript. All authors have read and approved the final version of this paper.

## Appendix

Variance components in FIA with dominance included (i.e. eq. 10) were estimated using the Fisher scoring algorithm given in Rönnegård and Carlborg [17].

For simulations under Case 1, the additive variance  and the covariance within lines  were similar, and the dominance variance  was close to the dominance covariance within lines  [see Additional File 1]. These results were expected since the correlation within lines is 1.0 in Case 1. Furthermore, the relative difference between the estimated variances and covariances increased when the simulated within-line correlation decreased from 1.0 in Case 1 to 0 in Case 4.

The theoretical expectation of the estimated  and  for fixed values of *a *and *d *depends on the level of fixation within lines (see Appendix in Rönnegård et al. [3]). For a given case in Table A1 we can see, however, that the estimated QTL variances decreases as the simulated QTL effects decreases. For *a *= 0 or *d *= 0 we do not get QTL variance estimates close to zero, which suggests that there is a bias in the estimates. This bias is likely due to the fact that the elements in the IBD matrices Π and Δ are correlated, and that it is therefore difficult to separate the additive and dominance effects in the REML estimation. In the applied Fisher scoring algorithm, each variance component was restricted to be greater or equal to 0.1 to ensure positive variance estimates. If the algorithm had not converged within 20 iterations the result was not analyzed and reported as non-converged. There are five variance components in eq. (10) and there were a substantial number of simulations (around 15%) that did not converge. The difficulties in convergence is not a major problem in FIA, however, since the genome scan is based on a score statistic that does not require VC estimation. REML estimation for models with several variance components is a general computational problem and a robust method is described in Mishchenko et al. [12].

## Supplementary Material

Additional file 1 **Variance Component Estimation**. Table including variance component estimation for the FIA model with dominance included. Click here for file
