# Supplementary material for: Modelling dominance in a flexible intercross analysis
Source: BMC Genet. 2009 Jun 28;10:30. doi: 10.1186/1471-2156-10-30 (PMC2716366; doi:10.1186/1471-2156-10-30)
Supplement: Additional file 1 — Variance Component Estimation. Table including variance component estimation for the FIA model with dominance included. [file 1471-2156-10-30-S1.pdf]

Table A1 - Variance component estimation

Estimated variance components from the FIA model in eq.(10). 120 replicates were produced for each of the four simulated cases (see Table 1) and for three combinations of additive (a) and dominance effects (d). A pedigree with four founders and 800 F_2 individuals was simulated. A residual variance of 98 was simulated in all replicates. Mean values of variance component estimates are given together with standard errors as subscripts. The number of estimates that did not converge within 20 iterations are also presented.

		σ_v^2	σ_{c_1}	σ_d^2	σ_{c_2}	σ_e^2	Non-converged
$a = 2$ and $d = 0$	Case 1	$4.00_{0.24}$	$3.72_{0.23}$	$0.31_{0.06}$	$0.19_{0.02}$	$96.81_{0.45}$	33
	Case 2	$4.00_{0.24}$	$3.72_{0.23}$	$0.41_{0.05}$	$0.23_{0.03}$	$98.19_{0.51}$	11
	Case 3	$2.84_{0.16}$	$1.37_{0.12}$	$0.57_{0.08}$	$0.21_{0.03}$	$98.14_{0.46}$	6
	Case 4	$2.12_{0.11}$	$0.14_{0.02}$	$0.49_{0.09}$	$0.21_{0.03}$	$97.80_{0.47}$	5
$a = 1$ and $d = 1$	Case 1	$1.72_{0.19}$	$1.63_{0.17}$	$1.73_{0.19}$	$1.40_{0.16}$	$96.91_{0.55}$	32
	Case 2	$2.00_{0.16}$	$1.44_{0.14}$	$1.24_{0.14}$	$0.72_{0.09}$	$97.35_{0.47}$	23
	Case 3	$2.05_{0.14}$	$1.30_{0.13}$	$1.14_{0.13}$	$0.42_{0.07}$	$96.42_{0.54}$	19
	Case 4	$0.93_{0.08}$	$0.14_{0.02}$	$0.84_{0.09}$	$0.18_{0.02}$	$98.15_{0.52}$	6
$a = 0$ and $d = 2$	Case 1	$0.14_{0.02}$	$0.10_{0.00}$	$3.34_{0.23}$	$2.96_{0.21}$	$98.07_{0.41}$	18
	Case 2	$0.19_{0.03}$	$0.15_{0.02}$	$1.90_{0.16}$	$1.36_{0.13}$	$98.36_{0.47}$	10
	Case 3	$0.32_{0.05}$	$0.24_{0.03}$	$1.72_{0.17}$	$0.63_{0.10}$	$98.51_{0.48}$	11
	Case 4	$0.28_{0.05}$	$0.19_{0.02}$	$1.37_{0.12}$	$0.25_{0.04}$	$97.14_{0.48}$	14
